# Upward Lightning at the Gaisberg Tower: The Larger‐Scale Meteorological Influence on the Triggering Mode and Flash Type

**DOI:** 10.1029/2022JD037776

**Published:** 2023-05-23

**Authors:** Isabell Stucke, Deborah Morgenstern, Gerhard Diendorfer, Georg J. Mayr, Hannes Pichler, Wolfgang Schulz, Thorsten Simon, Achim Zeileis

**Affiliations:** ^1^ Institute of Statistics University of Innsbruck Innsbruck Austria; ^2^ Institute of Atmospheric and Cryospheric Sciences University of Innsbruck Innsbruck Austria; ^3^ Department of ALDIS (Austrian Lightning Detection and Information System) OVE Service GmbH Vienna Austria; ^4^ Department of Mathematics University of Innsbruck Innsbruck Austria

**Keywords:** self‐initiated upward lightning, upward lightning detectability, Gaisberg, ICC only, random forest, meteorological reanalysis

## Abstract

Upward lightning is rarer than downward lightning and requires tall (100+ m) structures to initiate. It may be either self‐initiated or triggered by other lightning discharges. While conventional lightning location systems (LLSs) detect most of the upward lightning flashes superimposed by pulses or return strokes, they miss a specific flash type that consists only of a continuous current. Globally, only few specially instrumented towers can record this flash type. The proliferation of wind turbines in combination with damages from upward lightning necessitates an improved understanding under which conditions self‐initiated upward lightning and the continuous‐current‐only subtype occur. This study uses a random forest machine learning model to find the larger‐scale meteorological conditions favoring the occurrence of the different phenomena. It combines ground truth lightning current measurements at the specially instrumented tower at Gaisberg mountain in Austria with variables from larger‐scale meteorological reanalysis data (ERA5). These variables reliably explain whether upward lightning is self‐initiated or triggered by other lightning discharges. The most important variable is the height of the −10°C isotherm above the tall structure: the closer it is, the higher is the probability of self‐initiated upward lightning. For the different flash types, this study finds a relationship to the larger‐scale electrification conditions and the LLS‐detected lightning situation in the vicinity. Lower amounts of supercooled liquid water, solid, and liquid differently sized particles and no LLS‐detected lightning events nearby favor the continuous‐current‐only subtype compared to the other subtypes, which preferentially occur with LLS‐detected lightning events within 3 km from the Gaisberg Tower.

## Key Points

- The closer the thundercloud’s main electrification region to the tower, the higher the probability that upward lightning is self‐initiated
- Continuous‐current‐only flashes have network‐detected lightning nearby in only 37% of upward lightning events, the other types in 92%
- Larger‐scale meteorological conditions let a random forest model distinguish upward lightning flash types with an area under the receiver operating characteristic curve of 0.75

## 1 Introduction

Upward lightning (UL) initiated from the Earth surface extending toward the clouds is much rarer than downward lightning initiated within the clouds extending toward the ground. Nevertheless, UL might pose a much larger damage potential as it is capable of transferring large amounts of charge up to hundreds of coulombs within a comparably long period of time (e.g., Birkl et al., 2017; Diendorfer et al., 2015). Tall structures (on the order of 100 m) are preferred starting locations for UL (e.g., Rakov & Uman, 2003). Wind turbines typically exceed such heights and consequently lightning damages to them have gone up hand in hand with their ever‐growing number in the quest for renewable energy sources (e.g., Birkl et al., 2018; Montanyà Puig et al., 2016; Pineda, Montanyà, Romero, et al., 2018; Pineda, Montanyà, Salvador, et al., 2018; Rachidi et al., 2008). Therefore, a comprehensive risk assessment of UL to wind turbines and other tall structures becomes increasingly relevant.

Proper risk assessment is, however, crucially impeded as ground truth lightning current measurements indicate that more than 50% of UL might not be detected by lightning location systems (LLSs, Diendorfer et al., 2015). LLS reliably detects other lightning types over larger regions, but not lightning that consists only of a relatively low‐amplitude constant initial continuous current (ICC_only_, e.g., Birkl et al., 2018). LLS requires the fast rising current in the lightning channel to emit sufficiently large electromagnetic field pulses to be detected (Diendorfer et al., 2009). Consequently, using only LLS observations might substantially underestimate the risk of lightning damage (e.g., Rachidi et al., 2008; Smorgonskiy et al., 2011). Globally, only few specially instrumented towers exist that can measure the ICC_only_ UL flash type.

**Figure 1:**
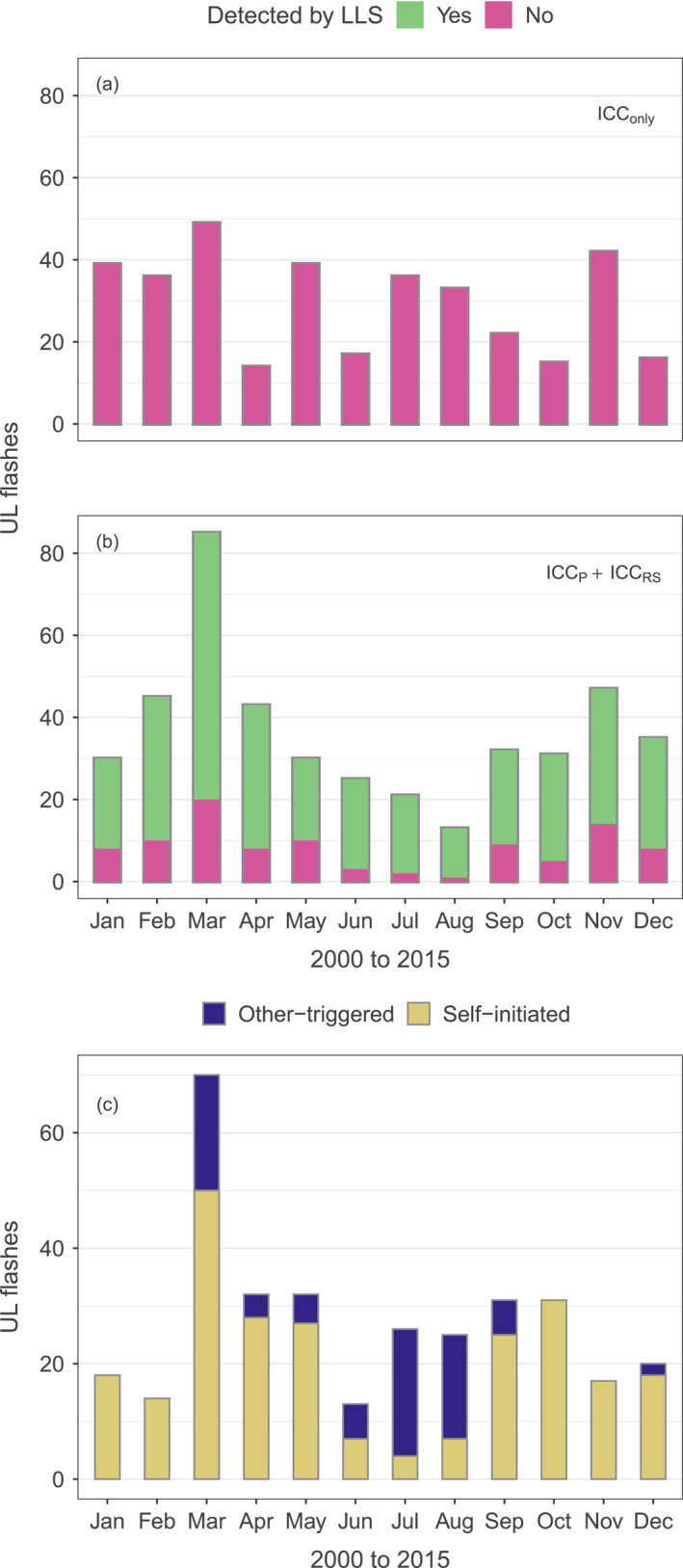
(a, b) Number of upward lightning (UL) observed at the Gaisberg Tower (2000–2015: total of 790 UL events) split into two categories. (a) Initial continuous‐current‐only subtype (ICC_only_). (b) Sum of UL with initial continuous current superimposed by pulses and by return‐stroke sequences, respectively (ICC_P_ + ICC_RS_). Colors distinguish detection (green) by the European Cooperation for Lightning Detection (EUCLID) lightning location system (LLS) from nondetection (red). (c) Number of UL from Gaisberg Tower classified as self‐initiated (yellow) and other‐triggered (blue) following the classification scheme by Zhou et al. (2012). Based on 329 observations from 2000 to 2015.

One of these towers with a height of 100 m is on top of the Gaisberg mountain in Austria. There, almost all lightning strikes are of the upward type (Diendorfer et al., 2015). Figure 1a shows that none of the ICC_only_ subtype could be detected by the regional LLS. According to Diendorfer et al. (2015), the other two subtypes of UL presented in Figure 1b, namely ICC followed by return strokes (ICC_RS_) and ICC superimposed by pulses (ICC_P_), were detected by the LLS in 96% and 58% of the cases, respectively. ICC_P_ type flashes are upward discharges with an ICC not followed by any return strokes but superimposed by one or more ICC pulses with a pulse peak current >2 kA. The pulses of the ICC_P_ flash type are suggested to be a consequence of the leader/return‐stroke mode of charge transfer as highlighted by Azadifar et al. (2016). While geographical and local topographical effects have been suspected to influence various UL parameters as emphasized by March (2015) and Birkl et al. (2018), to our knowledge no systematic analysis has been conducted to find what influences the occurrence of the ICC_only_ UL flash type.

UL requires extremely high field intensities immediately above the ground or an object on it for a discharge to start (Rakov & Uman, 2003). Structures with an effective height of 500 m or more are assumed to deform the electric field sufficiently to experience only UL. In this context, another type of UL triggered by a rocket which is connected to the ground via wire has been comprehensively investigated. The benefit of predictability of rocket‐triggered over tower‐initiated UL has led to various studies conducted in the US (e.g., Bagheri et al., 2019; Kereszy et al., 2022; Miki et al., 2005; Winn et al., 2021) and in China (e.g., Fan et al., 2021; Jiang et al., 2020; Li et al., 2021). The properties of tower‐initiated and rocket‐triggered UL have been found to be very similar. However, tower‐initiated UL may experience two different modes. For shorter objects, other discharges (cloud‐to‐ground and intracloud) close by can deform the electric field sufficiently to trigger a so‐called “other‐triggered” upward leader. However, UL can also be initiated from the tall structure itself (“self‐initiated”).

Location, for example, being situated on an isolated hill, and meteorological conditions are assumed to be favorable ingredients for the occurrence of UL in general (e.g., Schumann et al., 2015) and probably also for the occurrence of self‐initiated UL, but details are not clear yet. Regional and seasonal differences are large. During six warm seasons at tower locations in the USA and Brazil, only other‐triggered UL occurred (Schumann et al., 2019). In other locations and for other studies, the ratio between self‐initiated and other‐triggered UL varies widely—1:1 in Japan in six winter seasons (Wang & Takagi, 2012). In the USA, Warner et al. (2012) show that the ratio is 1:4; almost 80% of self‐initiated lightning and 15% of other‐triggered lightning occurred in the cold season. In Germany from measurements at the Peissenberg Tower, Manhardt et al. (2012) infer a ratio of 9:1; all self‐initiated UL occurred in the cold season. At the Gaisberg Tower, the ratio is 3:1 as shown in Figure 1c.

In addition to site‐specific conditions (e.g., Smorgonskiy et al., 2015), differences in meteorological conditions have been proposed to explain variations of this ratio (Jiang et al., 2014; Mostajabi et al., 2018; Pineda et al., 2019; Smorgonskiy et al., 2015; Wang & Takagi, 2012; Yuan et al., 2017; Zhou et al., 2014). The results have been partly contradictory. For example, Zhou et al. (2014) and Smorgonskiy et al. (2015) found no significant relationship between the ambient wind speed and self‐initiated UL at Gaisberg, while Mostajabi et al. (2018) underline its importance both at Gaisberg and at Säntis (Switzerland). Temperature has a significant impact at the Gaisberg Tower (e.g., Zhou et al., 2014) and at the Säntis Tower (e.g., Pineda et al., 2019; Smorgonskiy et al., 2015), whereas wind speed is more crucial in studies conducted in Japan (Wang & Takagi, 2012), China (Yuan et al., 2017), and in the United States (Warner et al., 2014).

Improving the understanding of the triggering modes of UL and the occurrence of the ICC_only_ flash type from a general meteorological perspective and finding widely available proxies from which their existence can be deduced might be a first step toward a proper assessment of the risk of UL to tall structures.

The main objective of this study is to assess whether a comprehensive set of larger‐scale meteorological variables may explain when UL is self‐initiated and when UL only consists of an ICC. Recent advances make the objective of this study achievable: the availability of hourly and vertically highly resolved reanalyses of atmospheric conditions (ERA5, Hersbach et al., 2020) and powerful flexible machine learning techniques, which allow to combine them with the lightning current measurements.

This article is organized as follows: first, a brief overview of the data used is given including meteorological reanalysis data and lightning observations (Section 2). Next, the procedures and statistical approaches for the two stated issues are introduced in Section 3. The results in Section 4 are divided into three subparts from which each comprises both explorative and quantitative findings as well as a subsequent discussion. The first subpart (Section 4.1) presents and discusses the results for the triggering mode, the second subpart (Section 4.2) presents and discusses the results for the UL flash type and an additional analysis in the third subpart investigates the relationship between the presence of LLS‐detected lightning events in the vicinity and the UL flash type (Section 4.3). Finally, Section 5 summarizes the key findings.

## 2 Data

The study combines three different data sources. It uses larger‐scale meteorological reanalysis data on hourly resolution (ERA5, Hersbach et al., 2020). In addition, two variables are introduced to complement the climatological background reflecting atmospheric differences among seasons and among daytime. These are the day of the year and the hour of day. Further, ground truth lightning current measurements at the Gaisberg Tower in Salzburg (Austria) and LLS data measured remotely by the European Cooperation for Lightning Detection (EUCLID, Schulz et al., 2016) are used.

### 2.1 Atmospheric Reanalysis

ERA5 is ECMWF’s fifth generation of global climate reanalysis from 1950 onward. ERA5 has a spatial resolution of 31 km horizontally (available at a 0.25° × 0.25° latitude–longitude grid) and 137 levels vertically at hourly resolution. This study considers the lowest 74 levels extending to approximately 15 km altitude, well into the stratosphere.

Convection results from various processes acting on different scales down to microscales which reanalysis data are not able to resolve. Nevertheless, this study aims to find drivers for the UL flash type and triggering mode, however, from larger‐scale meteorology. For this purpose, the systematic search of meteorological variables is expanded beyond common convection proxies to a comprehensive set of variables to involve various processes that might be influential. Thus in total, 75 directly available and derived variables at the surface, on model levels, and integrated vertically are spatially and temporally bilinearly interpolated to each Gaisberg Tower observation of UL. The meteorological variables fall into five broad categories: cloud physics, temperature field, moisture field, surface exchange, and wind field. Grouping the large set of variables into broad subgroups shall facilitate the interpretation. A complete list of the variable groups and individual variables can be found in Supporting Information S1.

### 2.2 Lightning Observations

Since 1998, lightning currents have been directly measured at the Gaisberg Tower in Salzburg (Austria, Diendorfer et al., 2009). The 100‐m‐tall radio tower is situated on top of the Gaisberg mountain 1,288 m above mean sea level (47°48′N, 13°60′E). This study uses observations from 2000 to 2015 and is restricted to negative UL flashes only, as these make up the largest proportion (93%) and thus provide a consistent database. In total, 819 negative UL flashes were recorded at Gaisberg Tower during this period.

The identification of self‐initiated versus other‐triggered UL flashes relies on additional records of the transient electrical field at the tower site during UL initiation. Out of 819 UL flashes, 329 could be clearly identified as either self‐initiated or other‐triggered. In total, 329 UL events were investigated with respect to the ambient electrical field during UL initiation. This electrical field was measured by a flat plate antenna installed at a platform close to the Gaisberg Tower. If a rapid change in the transient field in response to a lightning discharge in the vicinity was observed just prior to the initiation of UL at the Gaisberg Tower, the event was flagged as other‐triggered lightning. If the rapid change in the transient field started just at the time of the initiation of UL at the Gaisberg Tower, it was flagged as self‐initiated UL. Further details can be found in Zhou et al. (2012).

The identification of the UL flash type exclusively relies on the direct current measurements at the top of the Gaisberg Tower. On the tower, a current‐viewing shunt resistor allows to measure the overall current waveform of an UL flash (see Diendorfer et al., 2009 for details). While all UL flashes develop an ICC, the overall waveform can be differentiated by three different characteristics leading to three distinct UL flash types. An ICC_RS_ type UL flash is characterized by a short phase after the ICC with no current followed by one or more return‐stroke‐like sequences with amplitudes commonly larger than 2 kA. An ICC_P_ type UL flash is characterized by an ICC with one or more pulses superimposed mostly stronger than 2 kA. ICC_only_ type UL does not have ICC pulses with amplitudes larger than 2 kA nor return strokes, which implies that LLS networks such as EUCLID cannot detect them.

Seven hundred and ninety UL observations could be unambiguously assigned to an UL flash type. Since the temporal resolution of the meteorological data is 1 hr, only events with the same UL flash type in 1 hr will be used, which leaves 403 UL flashes.

Finally, to investigate the overall larger‐scale lightning activity, LLS data are used. EUCLID measures downward lightning with a detection efficiency of more than 90% (Schulz et al., 2016). Intracloud discharges are detected with a quite variable efficiency ranging from 10% to 67% depending on the thunderstorm situation (Poelman & Schulz, 2020). The LLS measurements are used to determine whether UL was associated with any lightning nearby detected by the LLS. For this purpose, the closest lightning event (cloud‐to‐ground or intracloud) within a radial distance of up to 100 km and 1 min before or after an UL event is extracted as a proxy for the lightning activity detected by LLS.

## 3 Methods

An explorative analysis gives first insights whether there are variables, which are able to distinguish conditions yielding self‐initiated or other‐triggered UL and ICC_only_ or ICC_P_ + ICC_RS_ UL. The explorative analysis differentiates each category with respect to a larger‐scale meteorological variable. The larger the difference in the meteorological variable between the triggering modes and UL flash type categories, respectively, the higher the ability to separate the different groups from the larger‐scale meteorological setting.

The triggering mode and UL flash type occurrence might be highly nonlinearly related to the atmosphere represented by the 75 meteorological variables plus the two climatological background variables. To account for and quantify this relationship requires more sophisticated approaches. These quantitative approaches shall allow to assess (a) how well larger‐scale meteorology is capable of explaining the occurrence of the triggering modes and the different UL flash types, (b) which meteorological variables are important for the occurrence of a preferred triggering mode and UL flash type, and (c) the effect of influential variables on the triggering mode and UL flash type.

### 3.1 Statistical Modeling Through Random Forests

The quantitative analysis of this study relies on random forests. Random forests are a powerful machine learning technique used in various scientific fields (Strobl et al., 2009). They are extremely flexible and easy to set up while being very stable and typically with high diagnostic ability (Breiman, 2001). Random forests consist of individual trees (Hothorn et al., 2006). Regression and classification trees (Breiman et al., 1984) are popular models due to their flexibility and intuitive interpretation. Using an ensemble of many trees on random subsamples of the full input data substantially improves the performance over single trees.

Distinguishing the two different triggering modes and the UL flash type categories are binary classification problems. For the triggering modes, the response variable is self‐initiated versus other‐triggered UL; for the UL flash type, the response variable is ICC_only_ versus ICC_P_ + ICC_RS_ type UL. Seventy‐seven variables (cf. Section 2.1) are considered as explanatory variables. Ensembles of classification trees, that is, random forests shall capture the potential nonlinearities and interactions in the relationship between the two response variables and the explanatory variables. More specifically, conditional inference random forests (Hothorn et al., 2004) are used (Section 3.1) combined with permutation‐based variable importance measures to assess the relative influence of the different meteorological drivers (Section 3.2).

Each tree of a conditional random forest consists of nodes which are represented by so‐called split variables chosen based on permutation tests (also known as conditional inference, Strasser & Weber, 1999). Growing these individual trees comprises three steps. Finding an appropriate split variable from the set of meteorological variables, finding an appropriate threshold where to split in the respective split variable, and finding a point where to stop the tree growing. A simple example of a classification tree can be found in Supporting Information S1.

Each of these trees is based on a random subsample of the input data and is constructed in the following way. First, the meteorological variable with the strongest association is selected as split variable. Next, a reasonable split point in this split variable to separate the different response classes as well as possible is found using a permutation test statistic. Computing this test statistic over all possible subsets and setting the split point where it is most reasonable according to the test statistic allows to judge which threshold adds the most to the performance. The same variable and split point selection is then repeated recursively for all of the random subsamples of the input data. Splitting continues until a certain stopping criterion (e.g., significance or subsample size) is met. The random forest then averages the diagnosed probabilities from the ensemble of trees, which stabilizes and enhances the diagnostic performance. More details regarding the algorithm and corresponding implementation are provided in Hothorn et al. (2006) and Hothorn and Zeileis (2015).

### 3.2 Assessing the Variable Importance

Random forests greatly stabilize the inferred classification relationship and improve the diagnostic performance. One of the largest benefits using random forests is the ability to assess the importance of each variable for a successful classification. This is done with the permutation variable importance method (see Strobl et al., 2008). First, the values of each input variable are randomly mixed and second, it is computed how much this degrades the classification performance.

To assess this diagnostic performance, the variable importance is computed on data not used for modeling. The random forests are learned on two thirds of the input data and the permutation variable importance is assessed on the remaining one third of the input data (i.e., test data).

Diagnostic performance on the test data is described by a specific score. In this study, it is the area under the receiver operating characteristic curve (AUC, Wilks, 2011). AUC is a scalar value representing the ability to distinguish and hence correctly classify the response. The closer this value is to 1, the higher the performance. For the variable importance, a single driver variable is randomly permuted and the median AUC decrease, that is, the decrease in performance is computed across all test data sets. This decrease of diagnostic performance when permuting the indicated variable is computed by the normalized difference of the original sample score and the permuted sample score. The driver variable leading to the strongest decrease in the diagnostic performance score, that is, AUC is most important for the classification.

## 4 Results and Discussion

Larger‐scale meteorological conditions might play a crucial role in determining whether the tall Gaisberg Tower itself initiates UL and whether ICC_only_ UL occurs. The following section presents and discusses the explorative and quantitative results of the larger‐scale meteorological influence on the triggering modes and UL flash types. Further, the prevailing lightning activity is investigated for the different UL flash types using LLS data.

### 4.1 Triggering Modes of Upward Lightning

**Figure 2:**
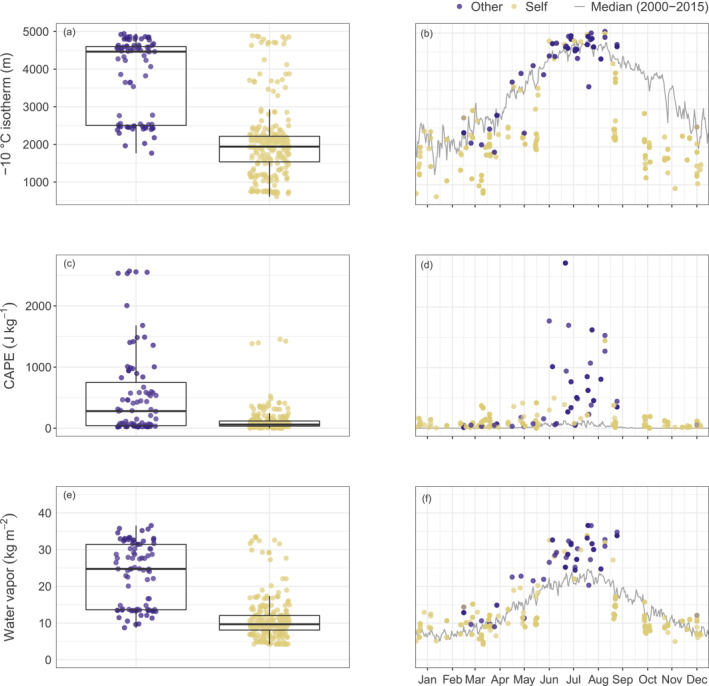
Distributions (left) and annual cycles (right) of three different larger‐scale meteorological variables during self‐initiated (yellow) and other‐triggered (blue) upward lightning (UL) events. Dots indicate values spatially and temporally interpolated to UL events at the Gaisberg Tower (329 UL events). Boxplots summarize the statistical distribution showing the median (vertical middle line), the interquartile range (IQR, 25%–75%), and whiskers (±1.5 × IQR). Gray lines in the right column panels illustrate the daily medians of the respective variable at the Gaisberg Tower from 2000 to 2015. (a, b) The −10°C isotherm height above ground, (c, d) the amount of convective available potential energy (CAPE), and (e, f) the total column water vapor.

In a first step, the statistical distributions of three variables explore the ability to separate events of self‐initiated UL from other‐triggered UL. The height of the −10°C isotherm is chosen since a proximity of the main charge separation region is likelier to induce UL directly at the tower. The more convective available potential energy (CAPE) is released, the more vigorous updrafts and thus charge separation can occur. And the more water vapor in the atmospheric column is available for conversion to solids, the more particles might become charged during collisions. The left column in Figure 2 distinguishes self‐initiated (yellow) and other‐triggered (blue) UL with respect to those three different larger‐scale meteorological variables.

Qualitatively, the −10°C isotherm height and water vapor may most clearly distinguish self‐initiated from other‐triggered UL (left column). Self‐initiated UL tends to occur at lower temperatures, at lower amounts of water vapor, and at lower amounts of CAPE compared to other‐triggered UL.

Since temperature‐related variables generally have a significant seasonal cycle, exploring the difference in the climatological distributions of influential variables for one or the other triggering mode gives further insights.

Strikingly, during self‐initiated UL events, the relevant variables in panels b and f do not follow a pronounced annual cycle and remain preferentially below the climatological median in winter and in the transition seasons. Contrarily, other‐triggered UL, which preferentially occurs in the warm season, tends to be close to or even above the daily median values.

Concerning CAPE in panel d, other‐triggered UL in the warm season occurs preferentially at higher CAPE values compared to self‐initiated UL, whereas self‐initiated UL occurs most often at a rather low or moderate amount of CAPE throughout the year.

While the connection between these three variables and UL can be physically explained and is clearly visible in the statistical distributions, further larger‐scale meteorological variables might be important and even interact nonlinearly. Therefore in a second step, random forest models with 75 larger‐scale meteorological plus time‐of‐day and day‐of‐year quantify the ability of meteorological conditions to separate self‐initiated from other‐triggered UL. Details of the model are given in Supporting Information S1.

The random forest models perform reliably and sharp (Gneiting et al., 2007). The AUC is at 0.93 very close to the optimum value of 1, which means that most of the distinction between self‐initiated and other‐triggered UL is based on larger‐scale meteorological conditions.

**Figure 3:**
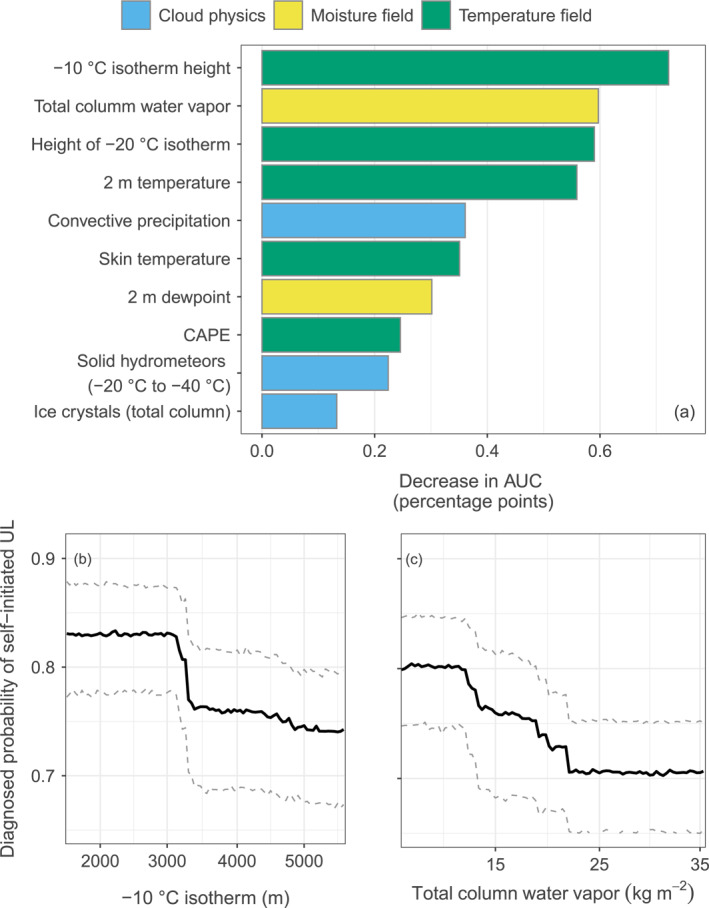
Ranking of the most important variables influencing whether upward lightning (UL) is self‐initiated according to the permutation variable importance procedure described in Section 3.2 (a). Colors indicate to which meteorological group the variables belong. Lower left (b) Effect of the −10°C isotherm height above ground on the probability of self‐initiated UL (value 1). Lower right (c) Effect of total column water vapor on the probability of self‐initiated UL. Dashed lines are the lower and upper bound of the 90% confidence interval indicating the uncertainty of the random forests.

The random forest models together with the variable importance are further able to identify which variables are most important for the differentiation of the triggering modes. The importance of the driving variables for the triggering mode is ranked in Figure 3a. Two of the variables in the previous exploratory analysis come out op—the height of the −10°C isotherm and total column water vapor. Five of the most important variables listed are part of the temperature field group. Particularly the height of the −10°C isotherm has a large influence on the self‐initiation of UL. Further, the −20°C isotherm height, the 2 m temperature, CAPE, and the skin temperature have an impact. Two additional variables strongly related to the temperature field are the total column water vapor and the 2 m dewpoint temperature from the moisture field group. The most influential variables from cloud physics are the convective precipitation, the proportion of solid hydrometeors between −20°C and −40°C, and ice crystals (total column).

The temperature‐related variables, the −10°C and −20°C isotherm height, 2 m temperature, skin temperature, 2 m dewpoint temperature, and water vapor in Figure 3 all share relatively high mutual information. Following the model fitting and the permutation variable importance process described in Section 3, different variables may be selected as potential splitting variables at different stages. If in one stage the most important variable, that is, the −10°C isotherm height is not selected, it might be replaced by one of the other variables serving as proxy for it.

Another important benefit of the conditional inference random forest models is that the effect of a single variable on the probability of self‐initiated UL or ICC_only_ UL can be assessed. The influence of a single variable in the random forests can be explained by varying it, while other explanatory variables are held constant at their mean value (panels b and c in Figure 3). The solid line shows the median diagnosed effects from the ensembles based on 100 learning samples including two thirds of the original number of observations. Varying the −10°C isotherm height, Figure 3b illustrates, that a higher −10°C isotherm decreases the probability of self‐initiated UL. The range between the dashed lines illustrates the confidence interval, that is, the uncertainty of the effect. Note a drop in the probability of self‐initiated UL associated with a −10°C isotherm between around 3,000 and 3,500 m above ground. This drop makes about seven percentage points in the probability of self‐initiated UL according to the median based on the random forests. Varying the total column cloud water vapor shows that a higher amount of water vapor decreases the probability of self‐initiated versus other‐triggered UL (panel c in Figure 3).

Results on the importance of temperature‐related variables for the occurrence of UL in general are in line with findings by other studies (e.g., Shindo et al., 2015). Most frequently, this importance is explained by the influence on the height of the cloud charge layer. Lower temperatures lower the cloud base and bring the main electrification region closer to the tall structure (Heidler et al., 2014). The reduced distance between the tall structure and the charged thundercloud then increases the electric field and eases the incidence of self‐triggered lightning flashes (e.g., Heidler et al., 2013; Pineda, Montanyà, Romero, et al., 2018; Pineda, Montanyà, Salvador, et al., 2018).

### 4.2 Flash Types of Upward Lightning

Whether larger‐scale meteorological conditions can distinguish as well between ICC_only_ UL events and ICC_P_ + ICC_RS_ UL events is investigated in this section. The first step is again an exploratory analysis with four potentially influential variables.

**Figure 4:**
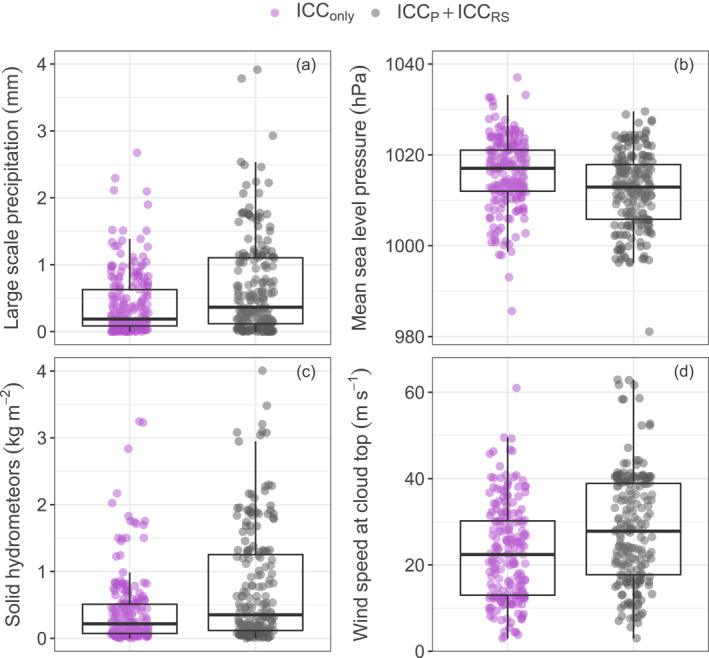
Distribution of relevant meteorological variables with respect to ICC_only_ type upward lightning (UL; purple) and ICC_P_ + ICC_RS_ type UL (gray). (a–d) Large‐scale precipitation, mean sea level pressure, the total column amount of solid hydrometeors, and the wind speed at cloud top. Dots are spatially and temporally interpolated to Gaisberg observations (403) and jittered vertically. Boxplots summarize the statistical distribution.

Figure 4 shows that the lower the amount of large‐scale precipitation, the proportion of solid hydrometeors, the wind speed at cloud top and the higher the mean sea level pressure, the higher the proportion of ICC_only_ UL over ICC_P_ + ICC_RS_ UL at the Gaisberg Tower. The separation between the two flash type categories is, however, weaker than the case of UL triggers (cf. Figure 2, left column). One important indicator about the complexity to clearly distinguish both UL flash type categories is that in 387 out of the 790 cases both flash type categories occur within the same hour. The larger‐scale meteorological data on hourly resolution can, however, only provide a clear separation when 1 hr contains either an ICC_only_ or an ICC_P_ + ICC_RS_ UL flash type. The random forest models based on the remaining 403 observations are, however, able to quantify the importance of particular larger‐scale meteorological conditions that preferentially yield one or the other flash type category. The reason is that random forests combine many explanatory variables having individually rather low influence, that is, weak learners, to a strong learner to yield a model from which information can be inferred (James et al., 2013). With an AUC of 0.75, the performance of the UL flash type classification models is lower than the performance of the triggering mode models, but still at a high value. Valuable information is carried by the random forest models with respect to influential variables distinguishing the larger‐meteorological setting when ICC_only_ UL or ICC_P_ + ICC_RS_ UL occurs.

**Figure 5:**
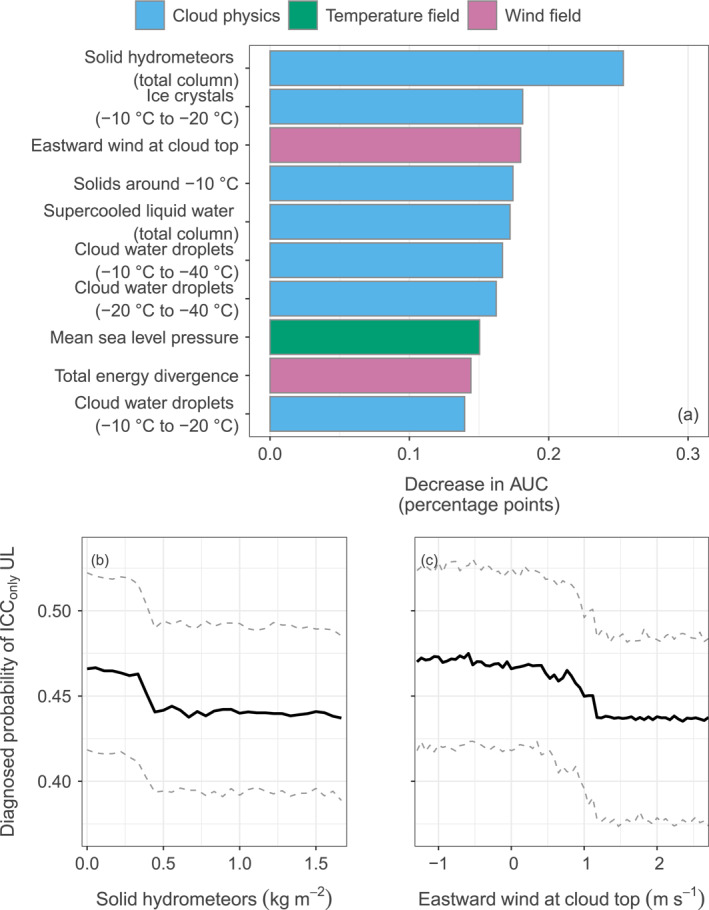
Ranking of the most important variables influencing whether upward lightning (UL) is of ICC_only_ type according to the permutation variable importance procedure described in Section 3.2 (a). Colors indicate to which meteorological group the variables belong. Lower left (b) Effect of solid hydrometeors (total column) on the probability of ICC_only_ UL (value 1). Lower right (c) Effect of the eastward wind at cloud top. Solid lines show the median effect; the dashed lines the lower and upper bound of the 90% confidence interval.

Cloud physics variables dominate the list of the 10 most influential variables when they are individually randomly permuted. Seven out of the 10 are cloud physics variables (see panel a in Figure 5). Permuting individual variables shows that the total column amount of precipitable solid hydrometeors has the largest influence on the probability of ICC_only_ UL over ICC_P_ + ICC_RS_ UL. Other important variables from cloud physics are the amount of ice crystals between −10°C and −20°C, solids around −10°C, the total amount of supercooled liquid water and the amount of cloud water droplets between −10°C and −40°C, −20°C and −40°C, and −10°C and −20°C.

The seven variables from the cloud physics group in Figure 5a are key variables for the theory of charge separation (e.g., Williams, 2018). The charge separation process requires the presence of both supercooled liquid water and frozen particles. In the presence of supercooled liquid water, differently large frozen particles collide receiving a different polarity. Gravity‐driven motions then ensure the separation of differently charged particles resulting in a typical vertical charge structure. This charge structure is most often characterized by a dipole with a negative charge layer below a positive charge layer (see e.g., Williams, 2018). Tan et al. (2016) emphasize the importance of the dipole structure for the initiation of (negative) UL from tall structures as a consequence of the specific temperature stratification as well as the lowering of hydrometeors.

Another important group is the wind field, with the eastward component of the wind at cloud top and the total energy divergence. Stucke et al. (2022) demonstrate that UL at the Gaisberg Tower most frequently occurs associated with increased amounts of particles and hydrometeors and enhanced wind field variables.

From the temperature field, the mean sea level pressure and the SE‐NW pressure difference are among the 10 most influential variables.

The advantage using random forests to improve the relatively low power to distinguish the UL flash type of a single variable by combining many different variables can be observed in the effect of individual variables. Varying the total amount of solid hydrometeors shows that a higher amount decreases the probability of ICC_only_ type UL (panel b of Figure 3). According to the median, this probability decreases from about 0.47 to 0.43 when the amount of solid hydrometeors changes from 0 kg m^−2^ to more than 1.5 kg m^−2^. A similar, relatively small effect can be observed for all the other variables related to cloud physics in the list. The most important wind field variable, the eastward component of the wind at cloud top also shows a decreasing probability with increasing wind velocities toward the east (panel c of Figure 3).

Even though the data used in this study are on significantly larger scales compared to the microphysical scale on which charge separation happens, the random forests identify the importance of the cloud physical variables. The effect of these variables is negative, meaning that conditions with smaller amounts of hydrometeors, supercooled liquid water, solid and liquid particles as well as weaker larger‐scale westerly winds at the cloud top favor the occurrence of ICC_only_ UL over ICC_P_ + ICC_RS_ UL. Weaker motion fields and fewer available particles might lead to fewer charge‐separating collisions and thus weaker electrification. Contrarily, a stronger (westerly) wind field that is capable to move a larger amount of solid and liquid particles as well as supercooled liquid water might cause stronger electrification conditions by enhanced charge separation compared to conditions favoring ICC_only_ UL.

Especially in the transition seasons and in winter, the atmosphere tends to be highly variable and influenced by the eastward progression of cyclones (Perry, 1987). The development and progression of midlatitude cyclones provides favorable conditions for so‐called wind‐field thunderstorms (Morgenstern et al., 2022). This thunderstorm type is among others associated with high wind speeds and shear, large amounts of precipitation as well as low CAPE. The importance of the eastward component of the wind instead of wind speed at the cloud top might be due to the specific topographic location of the Gaisberg Tower which lies at the W‐E oriented northern rim of the Alps. Westerly flow is thus unobstructed, whereas flow from the South is frequently blocked, deflected, or at least significantly slowed down by the Alpine topography.

As reanalysis data alone do not allow any further physical interpretation on the electrification and convective processes, the following section investigates the product of these—lightning events in the vicinity of the tower.

### 4.3 Nearby Lightning Events Detected by LLS

Meteorological processes on many scales—from the larger synoptic scale to mesoscale to cloud‐particle scales—lead to charge separation and subsequently to the development of thunderstorms. The presence of lightning events nearby might give valuable information on these processes not directly represented by larger‐scale meteorology alone. Further, lightning in the vicinity might also modify the electric field and hence influence the occurrence of different UL flash types. To investigate whether a particular connection exists, the spatially and/or temporally closest lightning event to an UL flash at the Gaisberg Tower within a window of 60 s before and after an UL flash and a radial distance of 100 km is extracted from the LLS EUCLID and used as a proxy for the LLS‐detected lightning activity nearby. Due to the limited efficiency of the LLS to detect intracloud discharges, a significant proportion of discharges nearby might be not represented in the following analysis. Moreover, LLS cannot differentiate between downward lightning and UL. To avoid that LLS‐detected UL itself is counted as closest LLS‐detected event, flashes that occurred right after the UL event at the Gaisberg Tower (between >= 0 s and < 1 s) are excluded.

A first explorative analysis reveals that only 37% of ICC_only_ type UL is accompanied by nearby LLS‐detected lightning events, but 92% of ICC_P_ + ICC_RS_ UL is accompanied by LLS‐detected lightning events nearby.

**Figure 6:**
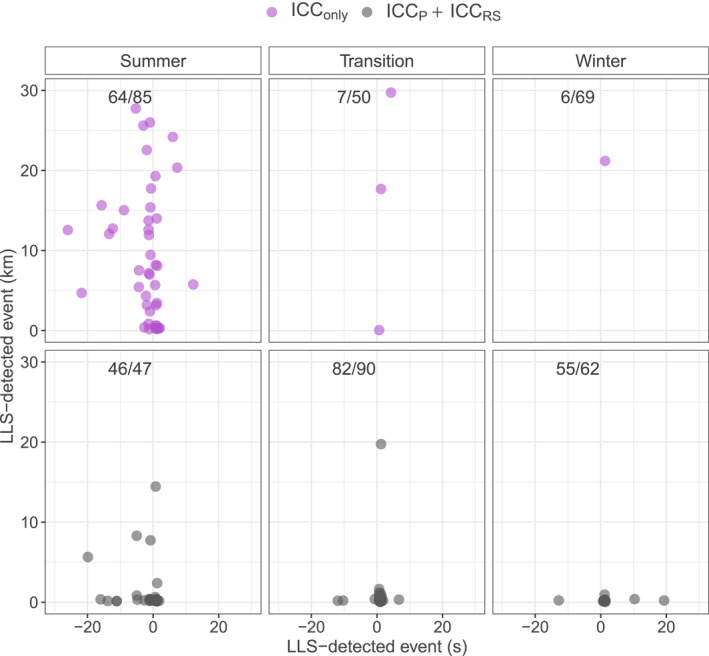
Distributions of spatial distances (*y*‐axis) of the closest lightning location system (LLS)‐detected lightning event within a time window (*x*‐axis) 10 s before (minus sign) and after upward lightning (UL) initiation at the Gaisberg Tower for ICC_only_ type UL (top row) and ICC_P_ + ICC_RS_ (bottom row), respectively. Based on the 403 events in which only one type occurred within a given hour. The three different panels in each row distinguish summer (MJJA), the transition seasons (SOMA), and winter (NDJF). The numbers in the left corner are the number of events with nearby LLS‐detected lightning events (closer than 100 km and 60 s before or after the UL event at the Gaisberg Tower)/the total number of UL events of the respective flash type in the respective season.

Given that there are LLS‐detected lightning events nearby, Figure 6 illustrates the distributions of the spatial (*y*‐axis) and temporal (*x*‐axis) distance of these nearby LLS‐detected events to the Gaisberg Tower with respect to the different UL flash type categories and with respect to the different seasons, summer (May–August), winter (November–February), and the transition seasons (March–April, September and October).

Figure 6 shows that ICC_only_ UL accompanied by LLS‐detected lightning events nearby occurs almost exclusively in summer. Further, the closest LLS‐detected lightning events do not preferentially occur within a specific distance from the Gaisberg Tower at UL initiation time but are distributed equally from below 1 km to more than 30 km. In the transition seasons and in winter, only 13 events are associated with nearby LLS‐detected lightning activity.

Contrarily, when ICC_P_ + ICC_RS_ UL occurs, the closest LLS‐detected lightning activity seems to occur within a preferred distance. Interestingly, this behavior is observed throughout the year. In the transition seasons and in winter, it is rarely more than about 3 km away from the Gaisberg Tower. In summer, it is rarely more than about 10 km, but again with a clear preference below 3 km from the Gaisberg Tower.

Random forest models allow to quantify the relationship of the nearby LLS‐detected lightning activity to the occurrence of a specific UL flash type. Three variables are added to the set of explanatory variables to indirectly account for the larger‐scale lightning activity: the spatial and the temporal distance of the closest LLS‐detected lightning event and the product of both. The additional explanatory variables shall both account for the situation in which no lightning events nearby have been detected by LLS and for the situation in which LLS‐detected lightning events occurred close to the Gaisberg Tower. This step requires transforming the explanatory variables appropriately. Exponential kernel functions with a normalization of the radial distance by 100 km, and temporal distance by 60 s before and after an UL flash are applied to them. Values approach 1 the closer LLS‐detected lightning events to the UL in space and time and approach 0 the further away from the Gaisberg Tower in space and time. A value of 0 indicates that there is no LLS‐detected lightning activity within 100 km and/or 60 s before or after the UL flash.

Results from the random forest models show that the improvement from including these explanatory variables is large when assessing the diagnostic performance. Including them increases the AUC from 0.75 to 0.91, which allows to reliably separate and hence diagnose the two UL flash type categories.

**Figure 7:**
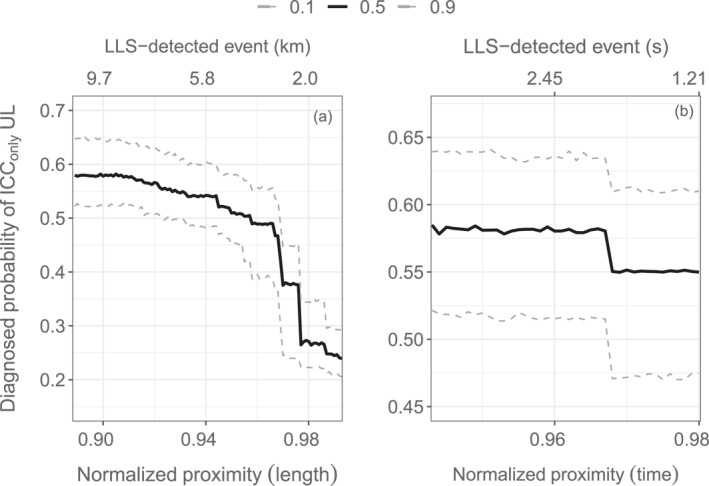
(a) Effect of lightning event distance in space on the probability of ICC_only_ upward lightning (UL; value 1). (b) Effect of the lightning event distance in time. Solid lines show the median effect; dashed lines are lower and upper bound of the 90% confidence interval.

The farther away (in space and time) the LLS‐detected lightning activity is, the higher the probability that the UL flash is of type ICC_only_ and not of type ICC_P_ + ICC_RS_ (Figure 7). The probability does not change gradually but jumps step‐like by more than 30 percentage points at a discharge distance from about 9 km to less than 500 m (a). The probability jumps at a discharge distance from about 1 to 2 s and has in total a weaker effect on the flash type.

While the statistical connection between nearby LLS‐detected nearby lightning activity and the types of UL flashes is clear, physical interpretations have to still be found.

One hypothesis is that ICC_only_ flashes occur preferably when conditions are not intense enough to produce natural downward lightning and enhanced LLS‐detected lightning activity. This might in general apply to situations with weak or no convection, for example, when charge‐separating motions result from the shear of the horizontal wind (Morgenstern et al., 2022) in the transitions seasons and in winter. It might also apply to the dissipating or early stages of thunderstorms. Compared to conditions yielding ICC_P_ + ICC_RS_ UL, all variables responsible for the electrification of a thundercloud are decreased when ICC_only_ UL occurs, that is, lower amounts of particles which might be charged and lower wind speeds and dynamic motions that trigger the collisions of particles. Further, ICC_only_ UL seems to occur most frequently when no nearby LLS‐detected lightning events are around or at least in summer when they are not preferably close to the Gaisberg Tower. This nevertheless does not imply that UL is a fair weather phenomenon.

Contrarily, 92% of ICC_P_ + ICC_RS_ UL occurs with LLS‐detected lightning events in the vicinity also and especially in winter and in the transition seasons. That the LLS‐detected lightning activity in general and the spatial distance of the closest LLS‐detected lightning event does not show a pronounced seasonal preference and that it is most frequently within a radius of about 3 km in winter and in the transitions seasons and most frequently less than 10 km away from the Gaisberg Tower in summer is in striking contrast to the occurrence of ICC_only_ UL. It appears that ICC_P_ + ICC_RS_ UL favors situations in which electrification is intense enough to produce LLS‐detectable downward lightning or intracloud discharges which might be in the developing or mature stage of a thunderstorm, for instance. The possibility of nearby lightning (LLS‐detectable or not) to trigger UL cannot be verified with the data used in this study.

## 5 Summary and Conclusions

This study investigates exploratively and quantitatively the larger‐scale meteorological impact on the triggering mode and UL flash type at the Gaisberg Tower. It applies random forests, a powerful and flexible machine learning technique, to a climatological data set of larger‐scale meteorological variables and ground truth lightning current measurements to diagnose (a) whether UL initiated from a tall tower is triggered by other discharges (“other‐triggered”) or by the tower itself (“self‐initiated”) and (b) of what particular flash type it is.

Ground truth lightning current measurements at the Gaisberg Tower in Austria between 2000 and 2015 are combined with 75 meteorological variables derived from the ERA5 reanalysis data set, time‐of‐day, day‐of‐year, and the occurrence of lightning in the vicinity detected by the EUCLID LLS. Whether UL is self‐initiated can be reliably explained by larger‐scale meteorological variables. The most important variable is the height of the −10°C isotherm above ground level. As the distance to the tall structure decreases, the probability of UL being self‐initiated increases. Further important variables are the −20°C isotherm height, the 2 m temperature, and CAPE. An important contribution comes further from variables of the cloud physics group such as water vapor, convective precipitation, and the proportion of solid hydrometeors between −20°C and −40°C and ice crystals. The lower the amount of water (gaseous, liquid, or solid), the higher the probability of self‐initiated UL.

Whether UL is of the ICC_only_ type has important consequences because that type cannot be spotted by LLSs but only by specially instrumented towers so that no regionally detailed information of their occurrence exists. An ICC_only_ type UL is characterized by an ICC only, whereas the other types have superimposed pulses or return stroke(s).

For the occurrence of ICC_only_ UL, the random forest models indicate that weaker electrification conditions with lower amounts of supercooled liquid water, hydrometeors, and particles in solid and liquid form and lower (westerly) wind speeds at cloud top yield more favorable conditions for ICC_only_ UL compared to ICC_P_ + ICC_RS_ UL.

However, explorative as well as quantitative examinations with random forests show that the larger‐scale meteorological conditions with additional information on the LLS‐detected lightning activity in the vicinity of the tower has an even stronger explanatory power to distinguish the UL flash types than considering the larger‐scale meteorological conditions alone. ICC_only_ type UL occurs most often when there is no LLS‐detected nearby lightning activity at all within 100 km and 60 s before or after the UL flash initiated from the tower. Further, mainly in summer ICC_only_, UL is associated with nearby LLS‐detected lightning activity, which has no preferred distance to the Gaisberg Tower up to several tenths of kilometers away.

Contrarily ICC_P_ + ICC_RS_ type UL occurs mainly associated with nearby LLS‐detected lightning activity, which has a preferred distance within about 3 km, quasi independent of the season. From this, it might be concluded that the intensity of the thunderstorm electrification due to seasonal differences or due to differences during electrification and the development of convection is influential on the UL flash type. This might imply that ICC_only_ UL occurs rather in weaker electrification conditions, in developing or dissipating convection and ICC_P_ + ICC_RS_ UL occurs rather in stronger electrification conditions (compared to ICC_only_ UL conditions) or in maturing convection. Hence, not only larger‐scale meteorological variables but additionally information on the electrification conditions must be taken into consideration to distinguish the meteorological conditions yielding ICC_only_ UL or ICC_P_ + ICC_RS_ UL.

## Conflict of Interest

The authors declare no conflicts of interest relevant to this study.

## Supporting information

Supporting Information S1

## Data Availability

ERA5 data are freely available at the Copernicus Climate Change Service (C3S) Climate Data Store (Hersbach et al., 2020). The results contain modified Copernicus Climate Change Service information (2020). Neither the European Commission nor ECMWF is responsible for any use that may be made of the Copernicus information or data it contains. EUCLID data and ground truth lightning current measurements from the Gaisberg Tower are available only on request. For more details, contact Wolfgang Schulz or Siemens BLIDS. *Software*. All calculations as well as setting up the final datasets, modeling, and the diagnosis were performed using R (R Core Team, 2021), using packages netCDF4 (Pierce, 2019), partykit (Hothorn & Zeileis, 2015), and ggplot2 package (Wickham, 2016). Retrieving the raw data and deriving further variables from ERA5 required using Python3 (Van Rossum & Drake, 2009) and CDO (Schulzweida, 2019).

## References

[jgrd58717-bib-0001] Azadifar, M. , Rachidi, F. , Rubinstein, M. , Paolone, M. , Diendorfer, G. , Pichler, H. , et al. (2016). Evaluation of the performance characteristics of the European lightning detection network EUCLID in the Alps region for upward negative flashes using direct measurements at the instrumented Säntis Tower. Journal of Geophysical Research: Atmospheres, 121, 595–606. 10.1002/2015JD024259

[jgrd58717-bib-0002] Bagheri, M. , Dwyer, J. R. , & McConnell, M. L. (2019). On the linear polarization of TGFS and X‐rays from natural and rocket‐triggered lightning and its association with source geometry. Journal of Geophysical Research: Space Physics, 124, 9166–9183. 10.1029/2019JA026570

[jgrd58717-bib-0003] Birkl, J. , Böhm, T. , Diendorfer, G. , Heidler, F. , Paul, C. , & Pichler, H. (2018). Measurement of lightning currents on high structures and wind turbines. In 2018 34th International Conference on Lightning Protection (ICLP) (pp. 1–8). IEEE. 10.1109/ICLP.2018.8503409

[jgrd58717-bib-0004] Birkl, J. , Shulzhenko, E. , Heidler, F. , & Diendorfer, G. (2017). Measuring lightning currents on wind turbines. Paper presented at 4th International Symposium on Winter Lightning (ISWL2017).

[jgrd58717-bib-0005] Breiman, L. (2001). Random forests. Machine Learning, 45, 5–32. 10.1023/A:1010933404324

[jgrd58717-bib-0006] Breiman, L. , Friedman, J. , Stone, C. J. , & Olshen, R. A. (1984). Classification and regression trees (Vol. 1). CRC Press. 10.1201/9781315139470

[jgrd58717-bib-0007] Diendorfer, G. , Pichler, H. , & Mair, M. (2009). Some parameters of negative upward‐initiated lightning to the Gaisberg Tower (2000–2007). IEEE Transactions on Electromagnetic Compatibility, 51(3), 443–452. 10.1109/TEMC.2009.2021616

[jgrd58717-bib-0008] Diendorfer, G. , Pichler, H. , & Schulz, W. (2015). LLS detection of upward initiated lightning flashes. In Proceeding of the 9th Asia–Pacific International Conference on Lightning (APL) (pp. 1–5).

[jgrd58717-bib-0009] Fan, Y. , Lyu, W. , Lu, G. , Wu, B. , Qi, Q. , Zhang, Y. , et al. (2021). Electromagnetic characteristics of upward leader initiated from the canton tower: A comparison with rocket‐triggered lightning. Journal of Geophysical Research: Atmospheres, 126, e2021JD034998. 10.1029/2021JD034998

[jgrd58717-bib-0010] Gneiting, T. , Balabdaoui, F. , & Raftery, A. E. (2007). Probabilistic forecasts, calibration and sharpness. Journal of the Royal Statistical Society, Series B: Statistical Methodology, 69(2), 243–268. 10.1111/j.1467-9868.2007.00587.x

[jgrd58717-bib-0011] Heidler, F. , Manhardt, M. , & Stimper, K. (2013). The slow‐varying electric field of negative upward lightning initiated by the Peissenberg Tower, Germany. IEEE Transactions on Electromagnetic Compatibility, 55(2), 353–361. 10.1109/TEMC.2012.2209121

[jgrd58717-bib-0012] Heidler, F. , Manhardt, M. , & Stimper, K. (2014). Self‐initiated and other‐triggered positive upward lightning measured at the Peissenberg Tower, Germany. In 2014 International Conference on Lightning Protection (ICLP) (pp. 157–166). 10.1109/ICLP.2014.6973113

[jgrd58717-bib-0013] Hersbach, H. , Bell, B. , Berrisford, P. , Hirahara, S. , Horányi, A. , Muñoz‐Sabater, J. , et al. (2020). The ERA5 global reanalysis. Quarterly Journal of the Royal Meteorological Society, 146(730), 1999–2049. 10.1002/qj.3803

[jgrd58717-bib-0014] Hothorn, T. , Hornik, K. , & Zeileis, A. (2006). Unbiased recursive partitioning: A conditional inference framework. Journal of Computational & Graphical Statistics, 15(3), 651–674. 10.1198/106186006X133933

[jgrd58717-bib-0015] Hothorn, T. , Lausen, B. , Benner, A. , & Radespiel‐Tröger, M. (2004). Bagging survival trees. Statistics in Medicine, 23(1), 77–91. 10.1002/sim.1593 14695641

[jgrd58717-bib-0016] Hothorn, T. , & Zeileis, A. (2015). partykit: A modular toolkit for recursive partytioning in R. Journal of Machine Learning Research, 16, 3905–3909.

[jgrd58717-bib-0017] James, G. , Witten, D. , Hastie, T. , & Tibshirani, R. (2013). An introduction to statistical learning (Vol. 103). Springer. 10.1007/978-1-4614-7138-7

[jgrd58717-bib-0018] Jiang, R. , Qie, X. , Li, Z. , Zhang, H. , Li, X. , Yuan, S. , et al. (2020). Luminous crown residual vs. bright space segment: Characteristical structures for the intermittent positive and negative leaders of triggered lightning. Geophysical Research Letters, 47, e2020GL088107. 10.1029/2020GL088107

[jgrd58717-bib-0019] Jiang, R. , Qie, X. , Wu, Z. , Wang, D. , Liu, M. , Lu, G. , & Liu, D. (2014). Characteristics of upward lightning from a 325‐m‐tall meteorology tower. Atmospheric Research, 149, 111–119. 10.1016/j.atmosres.2014.06.007

[jgrd58717-bib-0020] Kereszy, I. , Rakov, V. , Ding, Z. , & Dwyer, J. (2022). Ground‐based observation of a TGF occurring between opposite polarity strokes of a bipolar cloud‐to‐ground lightning flash. Journal of Geophysical Research: Atmospheres, 127, e2021JD036130. 10.1029/2021JD036130

[jgrd58717-bib-0021] Li, X. , Lu, G. , Jiang, R. , Zhang, H. , Fan, Y. , Shi, T. , et al. (2021). On the transition from precursors to the initial upward positive leader in negative rocket‐triggered lightning. Journal of Geophysical Research: Atmospheres, 126, e2020JD033926. 10.1029/2020JD033926

[jgrd58717-bib-0022] Manhardt, M. , Heidler, F. , & Stimper, K. (2012). The electric field of negative upward lightning strikes at the Peissenberg Tower, Germany. In 2012 International Conference on Lightning Protection (ICLP) (pp. 1–9). 10.1109/ICLP.2012.6344205

[jgrd58717-bib-0023] March, V. (2015). Upward lightning observations on a wind turbine and its implications to environmental factor for risk assessment. Paper presented at Proceedings of the Asia–Pacific Conference on Lightning Protection, Nagoya, Japan.

[jgrd58717-bib-0024] Miki, M. , Rakov, V. , Shindo, T. , Diendorfer, G. , Mair, M. , Heidler, F. , & Wang, D. (2005). Initial stage in lightning initiated from tall objects and in rocket‐triggered lightning. Journal of Geophysical Research, 110, D02109. 10.1029/2003JD004474

[jgrd58717-bib-0025] Montanyà Puig, J. , Fabró Tàpia, F. , Van der Velde, O. A. , March Nomen, V. , Williams, E. R. , Pineda Ruegg, N. , et al. (2016). Global distribution of winter lightning: A threat to wind turbines and aircraft. Natural Hazards and Earth System Sciences, 16(6), 1465–1472. 10.5194/nhess-16-1465-2016

[jgrd58717-bib-0026] Morgenstern, D. , Stucke, I. , Simon, T. , Mayr, G. J. , & Zeileis, A. (2022). Differentiating lightning in winter and summer with characteristics of the wind field and mass field. Weather and Climate Dynamics, 3(1), 361–375. 10.5194/wcd-3-361-2022

[jgrd58717-bib-0027] Mostajabi, A. , Sunjerga, A. , Azadifar, M. , Smorgonskiy, A. , Rubinstein, M. , Rachidi, F. , & Diendorfer, G. (2018). On the impact of meteorological conditions on the initiation of upward lightning flashes from tall structures. In 2018 34th International Conference on Lightning Protection (ICLP) (pp. 1–5). 10.1109/ICLP.2018.8503310

[jgrd58717-bib-0028] Perry, A. (1987). Middle latitude climates. In Climatology (pp. 581–583). Springer US. 10.1007/0-387-30749-4_116

[jgrd58717-bib-0029] Pierce, D. (2019). ncdf4: Interface to unidata netCDF (version 4 or earlier) format data files R package version 1.17 [Computer software manual]. Retrieved from https://CRAN.R-project.org/package=ncdf4

[jgrd58717-bib-0030] Pineda, N. , Figueras i Ventura, J. , Romero, D. , Mostajabi, A. , Azadifar, M. , Sunjerga, A. , et al. (2019). Meteorological aspects of self‐initiated upward lightning at the Säntis Tower (Switzerland). Journal of Geophysical Research: Atmospheres, 124, 14162–14183. 10.1029/2019JD030834

[jgrd58717-bib-0031] Pineda, N. , Montanyà, J. , Romero, D. , Bech, J. , Casellas, E. , & González, S. (2018). Meteorological aspects of winter upward lightning from an instrumented tower in the Pyrenees. In 2018 34th International Conference on Lightning Protection (ICLP) (pp. 1–7). 10.1109/ICLP.2018.8503271

[jgrd58717-bib-0032] Pineda, N. , Montanyà, J. , Salvador, A. , van der Velde, O. A. , & López, J. A. (2018). Thunderstorm characteristics favouring downward and upward lightning to wind turbines. Atmospheric Research, 214, 46–63. 10.1016/j.atmosres.2018.07.012

[jgrd58717-bib-0033] Poelman, D. R. , & Schulz, W. (2020). Comparing lightning observations of the ground‐based European lightning location system EUCLID and the space‐based lightning imaging sensor (LIS) on the international space station (ISS). Atmospheric Measurement Techniques, 13(6), 2965–2977. 10.5194/amt-13-2965-2020

[jgrd58717-bib-0034] R Core Team . (2021). R: A language and environment for statistical computing [Computer software manual]. Retrieved from https://www.R-project.org/

[jgrd58717-bib-0035] Rachidi, F. , Rubinstein, M. , Montanyà, J. , Bermudez, J.‐L. , Sola, R. R. , Sola, G. , & Korovkin, N. (2008). A review of current issues in lightning protection of new‐generation wind‐turbine blades. IEEE Transactions on Industrial Electronics, 55(6), 2489–2496. 10.1109/TIE.2007.896443

[jgrd58717-bib-0036] Rakov, V. A. , & Uman, M. A. (2003). Lightning: Physics and effects. Cambridge University Press. 10.1017/CBO9781107340886

[jgrd58717-bib-0037] Schulz, W. , Diendorfer, G. , Pedeboy, S. , & Poelman, D. R. (2016). The European lightning location system EUCLID—Part 1: Performance analysis and validation. Natural Hazards and Earth System Sciences, 16(2), 595–605. 10.5194/nhess-16-595-2016

[jgrd58717-bib-0038] Schulzweida, U. (2019). CDO user guide. Retrieved from 10.5281/zenodo.3539275

[jgrd58717-bib-0039] Schumann, C. , Saba, M. , Warner, T. , Ferro, M. , Helsdon, J. , Thomas, R. , & Orville, R. (2019). On the triggering mechanisms of upward lightning. Scientific Reports, 9(1), 9576. 10.1038/s41598-019-46122-x 31270371 PMC6610146

[jgrd58717-bib-0040] Schumann, C. , Saba, M. M. , de Paiva, A. , Jaques, R. , Schulz, W. , Diendorfer, G. , et al. (2015). Analysis of terrain and atmospheric conditions for upward flashes in Sao Paulo‐Brazil. In XIII International Symposium on Lightning Protection (SIPDA), Balneario Camboriu, Brazil (Vol. 28).

[jgrd58717-bib-0041] Shindo, T. , Miki, T. , Saito, M. , Asakawa, A. , Motoyama, H. , Ishii, M. , et al. (2015). Meteorological conditions and occurrence of upward lightning at high structures. IEEJ Transactions on Power and Energy, 135(6), 417–418. 10.1541/ieejpes.135.417

[jgrd58717-bib-0042] Smorgonskiy, A. , Rachidi, F. , Rubinstein, M. , Diendorfer, G. , & Schulz, W. (2011). On the proportion of upward flashes to lightning research towers. In 2011 7th Asia–Pacific International Conference on Lightning (pp. 858–862). 10.1109/APL.2011.6110248

[jgrd58717-bib-0043] Smorgonskiy, A. , Tajalli, A. , Rachidi, F. , Rubinstein, M. , Diendorfer, G. , & Pichler, H. (2015). An analysis of the initiation of upward flashes from tall towers with particular reference to Gaisberg and Säntis Towers. Journal of Atmospheric and Solar‐Terrestrial Physics, 136, 46–51. 10.1016/j.jastp.2015.06.016

[jgrd58717-bib-0044] Strasser, H. , & Weber, C. (1999). On the asymptotic theory of permutation statistics (Report Series SFB “Adaptive Information Systems and Modelling in Economics and Management Science” No. 27). SFB Adaptive Information Systems and Modelling in Economics and Management Science, WU Vienna University of Economics and Business. Retrieved from https://epub.wu.ac.at/102/

[jgrd58717-bib-0045] Strobl, C. , Boulesteix, A.‐L. , Kneib, T. , Augustin, T. , & Zeileis, A. (2008). Conditional variable importance for random forests. BMC Bioinformatics, 9(1), 307. 10.1186/1471-2105-9-307 18620558 PMC2491635

[jgrd58717-bib-0046] Strobl, C. , Malley, J. , & Tutz, G. (2009). An introduction to recursive partitioning: Rationale, application, and characteristics of classification and regression trees, bagging, and random forests. Psychological Methods, 14(4), 323–348. 10.1037/a0016973 19968396 PMC2927982

[jgrd58717-bib-0047] Stucke, I. , Morgernstern, D. , Diendorfer, G. , Mayr, G. J. , Pichler, H. , Schulz, W. , et al. (2022). Thunderstorm types and meteorological characteristics of upward lightning. In 2022 36th International Conference on Lightning Protection (ICLP) (pp. 282–288). 10.1109/ICLP56858.2022.9942489

[jgrd58717-bib-0048] Tan, Y. , Chen, C. , Zhou, J. , Zhou, B. , Zhang, D. , & Guo, X. (2016). A parameterization scheme for upward lightning in the cloud model and a discussion of the initial favorable environmental characteristics in the cloud. Science China Earth Sciences, 59(7), 1440–1453. 10.1007/s11430-016-5309-5

[jgrd58717-bib-0049] Van Rossum, G. , & Drake, F. L. (2009). Python 3 reference manual. CreateSpace Independent Publishing Platform.

[jgrd58717-bib-0050] Wang, D. , & Takagi, N. (2012). Characteristics of winter lightning that occurred on a windmill and its lightning protection tower in Japan. IEEJ Transactions on Power and Energy, 132(6), 568–572. 10.1541/ieejpes.132.568

[jgrd58717-bib-0051] Warner, T. , Cummins, K. , & Orville, R. (2012). Upward lightning observations from towers in Rapid City, South Dakota and comparison with National Lightning Detection Network data, 2004–2010. Journal of Geophysical Research, 117, D19109. 10.1029/2012JD018346

[jgrd58717-bib-0052] Warner, T. , Lang, T. J. , & Lyons, W. A. (2014). Synoptic scale outbreak of self‐initiated upward lightning (SIUL) from tall structures during the central US blizzard of 1–2 February 2011. Journal of Geophysical Research: Atmospheres, 119, 9530–9548. 10.1002/2014JD021691

[jgrd58717-bib-0053] Wickham, H. (2016). ggplot2: Elegant graphics for data analysis. Springer‐Verlag New York. Retrieved from https://ggplot2.tidyverse.org

[jgrd58717-bib-0054] Wilks, D. S. (2011). Statistical methods in the atmospheric sciences (Vol. 100). Academic Press.

[jgrd58717-bib-0055] Williams, E. (2018). Lightning activity in winter storms: A meteorological and cloud microphysical perspective. IEEJ Transactions on Power and Energy, 138(5), 364–373. 10.1541/ieejpes.138.364

[jgrd58717-bib-0056] Winn, W. , Trueblood, J. , Eack, K. , Edens, H. , Eastvedt, E. , Aulich, G. , et al. (2021). Triggered negative lightning‐leaders that propagated into thunderstorm lower positive charge. Journal of Geophysical Research: Atmospheres, 126, e2021JD034938. 10.1029/2021JD034938

[jgrd58717-bib-0057] Yuan, S. , Jiang, R. , Qie, X. , Wang, D. , Sun, Z. , & Liu, M. (2017). Characteristics of upward lightning on the Beijing 325 m meteorology tower and corresponding thunderstorm conditions. Journal of Geophysical Research: Atmospheres, 122, 12093–12105. 10.1002/2017JD027198

[jgrd58717-bib-0058] Zhou, H. , Diendorfer, G. , Thottappillil, R. , Pichler, H. , & Mair, M. (2012). Measured current and close electric field changes associated with the initiation of upward lightning from a tall tower. Journal of Geophysical Research, 117, D08102. 10.1029/2011JD017269

[jgrd58717-bib-0059] Zhou, H. , Diendorfer, G. , Thottappillil, R. , Pichler, H. , & Mair, M. (2014). The influence of meteorological conditions on upward lightning initiation at the Gaisberg Tower. In 2014 International Conference on Lightning Protection (ICLP) (pp. 1162–1165). 10.1109/ICLP.2014.6973303

