# Supplementary material for: Upward Lightning at the Gaisberg Tower: The Larger‐Scale Meteorological Influence on the Triggering Mode and Flash Type
Source: J Geophys Res Atmos. 2023 May 23;128(10):e2022JD037776. doi: 10.1029/2022JD037776 (PMC10909485; doi:10.1029/2022JD037776)
Supplement: Supplementary file 1 — Supporting Information S1 [file JGRD-128-e2022JD037776-s001.pdf]

Supporting Information for "Upward Lightning at the Gaisberg Tower: The Larger-scale Meteorological Influence on the Triggering Mode and Flash Type"

Isabell Stucke[1,2], Deborah Morgenstern[1,2],Gerhard Diendorfer[3], Georg J. Mayr[2], Hannes Pichler[3], Wolfgang Schulz[3], Thorsten Simon[4], Achim Zeileis[1]

[1]Institute of Statistics, University of Innsbruck, Austria, Innsbruck

[2]Institute of Atmospheric and Cryospheric Sciences, University of Innsbruck, Austria, Innsbruck

[3]OVE Service GmbH, Dept. ALDIS (Austrian Lightning Detection & Information System), Austria, Vienna

[4]Department of Mathematics, University of Innsbruck, Austria, Innsbruck

Contents of this file

1. Text Sections 1 to 3
2. Figures S1 to S5
3. Table S1

Introduction The supporting information comprises three key content categories. The first important information section introduces all the atmospheric variables which are available for selection during the modeling procedure of the random forests. These are either taken directly from the freely available ERA5 dataset at the Copernicus Climate

---

Corresponding author: I. Stucke, Institute of Statistics University of Innsbruck, Innsbruck, Universitätsstrasse 15, 6020, Austria. (isabell.stucke@uibk.ac.at)

Change Service (C3S) or derived from the variables available on different model levels, at the surface or integrated vertically. Variables are assigned to different meteorological groups: Cloud physics, moisture field, surface exchange, temperature field and wind field. Two additional terms shall represent the climatological background. All variables are available on a 31 km x 31 km grid and hence represent the larger-scale environment rather than the convective scale environment.

The second section illustrates an example of one classification tree being the basis of the random forest models. It shall demonstrate the splitting concept within the construction of a simple classification tree involving nodes, splitting thresholds and terminal nodes. One random forest model is built of 500 trees in this study.

The third part of the supporting information demonstrates the performance of the different random forest models in more detail. We focus on the general performance by analyzing the sharpness and reliability of the resulting models. In Sect. 3.1 the performance of the models classifying self-initiated and other-triggered upward lightning at the Gaisberg Tower is demonstrated. In Sect. 3.2 and Sect. 3.3 we present the performance of the models classifying the two different flash type categories: $\mathrm{ICC}_{\mathrm{only}}$ and $\mathrm{ICC}_{\mathrm{P}} + \mathrm{ICC}_{\mathrm{RS}}$ upward lightning.

1. Larger-scale Atmospheric Variables

The larger-scale atmospheric variables were selected and derived based on expert knowledge of the authors. To include as many processes as possible, not only variables related to convection from the larger-scale setting but variables representing many other processes from the five different meteorological groups are included. Each variable is bilinearly interpolated to each upward lightning event at the Gaisberg Tower becoming a predictor in

the problem classifying self-initiated over other-triggered or $\mathrm{ICC_{only}}$ over $\mathrm{ICC_P} + \mathrm{ICC_{RS}}$ upward lightning. Table S1 lists the atmospheric variables sorted by meteorological group.

2. Classification Tree

Figure S1 demonstrates the structure of a single classification tree. It shows several nodes with the particular split variables (e.g., the first split variable is the 2 m temperature). Between the single nodes, you see the thresholds, where the split variable is split for a best performance. Taking one upward lightning observation and following a certain path depending on the thresholds in the split variables ends in one of the terminal nodes which illustrate the classification in form of bars in this case. The colors of the bars indicate how many observations that end in the particular terminal node are self-initiated or other triggered.

3. Model Performance

3.1. Performance of Random Forests Classifying Self-Initiated versus Other-Triggered Upward Lightning

We present the performance of the random forests classifying self-initiated over other-triggered upward lightning in the following. Figure S2 shows that the random forest models built from the pool of 77 predictor variables are both relatively reliable (a) and sharp (b) when tested on unseen data samples. The 100 test data samples always include one-third of the original number of observations not considered during the training procedure of the ensembles. The value 1 is associated with a self-initiated flash and the value 0 is associated with an other-triggered flash. The error bars in (a) indicate the uncertainty of the predictions on the 100 test data samples by the 95 % confidence interval. The shaded area indicates the 95 % confidence interval of the average predicted probability.

The refinement distribution in (b) is based on accumulated predictions. For each bin the difference between the average observed relative frequency and the predicted probabilities for of the test data samples (dots) are around zero and mainly fall into the 95% confidence interval of the predicted probabilities indicating a reliable performance. The larger deviation from zero in the lower close to a 0 % probability of self-initiated flashes may attribute to the low number of predictions in this segment (see also in the refinement).

The predictions are not only reliable but also sharp as the concave like shape of the refinement curve in (b) and the maximum number of predictions close to the 100 % probability of self-initiated flashes (value 1) indicate. The median AUC value of 0.93 based on the test data samples underlines the ability to reliably separate self-initiated from other-triggered flashes from atmospheric variables.

3.2. Performance of Random Forests Classifying the Flash Type (Without Nearby Lightning Situation)

In this part we demonstrate the performance of the random forests classifying the flash type without considering the nearby lightning activity. Figure S3 depicts that the models are relatively well-calibrated and reliable up to predicted probabilities around 75 %. Both panels (a) and (b) can be interpreted analogously to Figure S2 from the previous section, whereas the value 1 is associated with $\mathrm{ICC_{only}}$ flashes and the value 0 is associated with $\mathrm{ICC_P + ICC_{RS}}$ flashes. The difference of the average observed relative frequency and the predictions according to the 100 test data samples including one-third of 403 observations lie within the 95 % confidence interval of the predictions below 75 %. However, both the refinement distribution (b) and the difference between observed and predicted probabilities (a) show that the models are not sharp. They most frequently predict in a probability

range between about 0.4 and 0.7 forming a hill shaped distribution. Further the models fail to correctly predict ICC_{only} flashes reflected by the large deviation of the average observed from the predicted probabilities close to 1, i.e., 100 %. The median in the AUC based on 100 test samples is 0.75, which is at a quite high value.

3.3. Performance of Random Forests Classifying the Flash Type (With Nearby Lightning Situation)

Including the nearby lightning activity in the previous classification problem clearly improves the results (Figure S4). ICC_{only} can now be more sharply separated from $ICC_P + ICC_{RS}$ flashes. Most frequently the model identifies the upward flash to be ICC_{only} with a probability of close to 90 % and 0 %, respectively. The difference between the average observed frequency and the predicted probabilities for each bin lie around zero and approach the zero line in the tails around 0 % and 90 %. The highest deviation from a zero difference is around predictions of 60 % which the models predict more rarely compared to the tails as the refinement in (b) demonstrates. This is in stark contrast to Figure S3 without nearby lightning information showing the smallest difference for the intervals from close to 0 % to about 75 % and the largest differences approaching the right tail at 100 %. The improvement of including the three variables to the dataset can be also seen in Figure S5. Losing the information of the discharge activity reduces the performance by more than 30%.

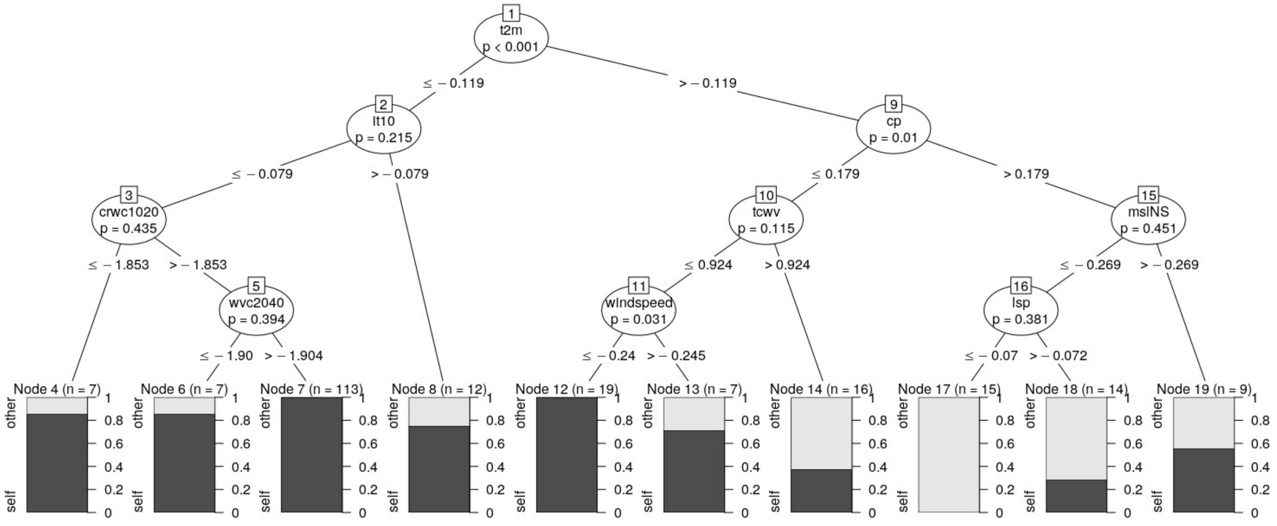

Figure S1. Example of a simple decision tree. Response variable is self-initiated versus other-triggered upward lightning. Nodes include the split variables and numbers are thresholds where the variable is split.

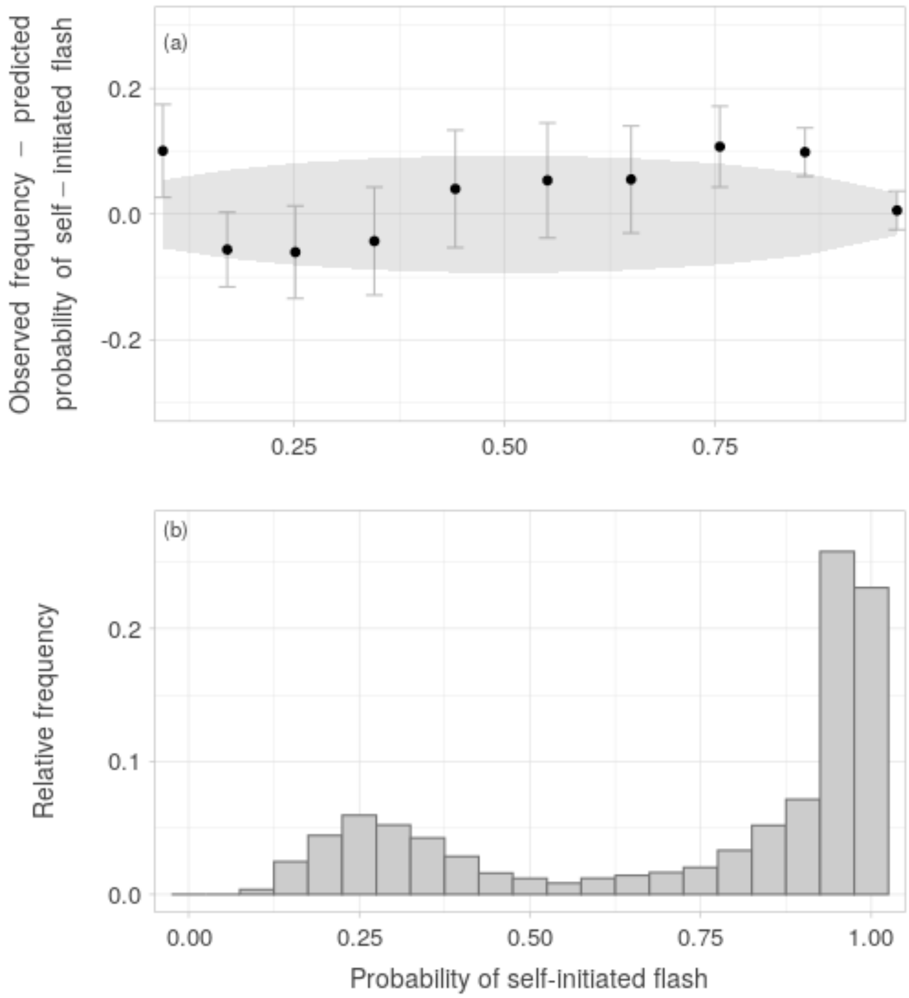

Figure S2. Panel (a): Difference of average observed relative frequency and predicted probability and refinement distribution (b) based on test data samples. Median of AUC is 0.93. Shaded area and error bars indicate the 95 % confidence interval around the average predicted probabilities and average observed frequencies, respectively.

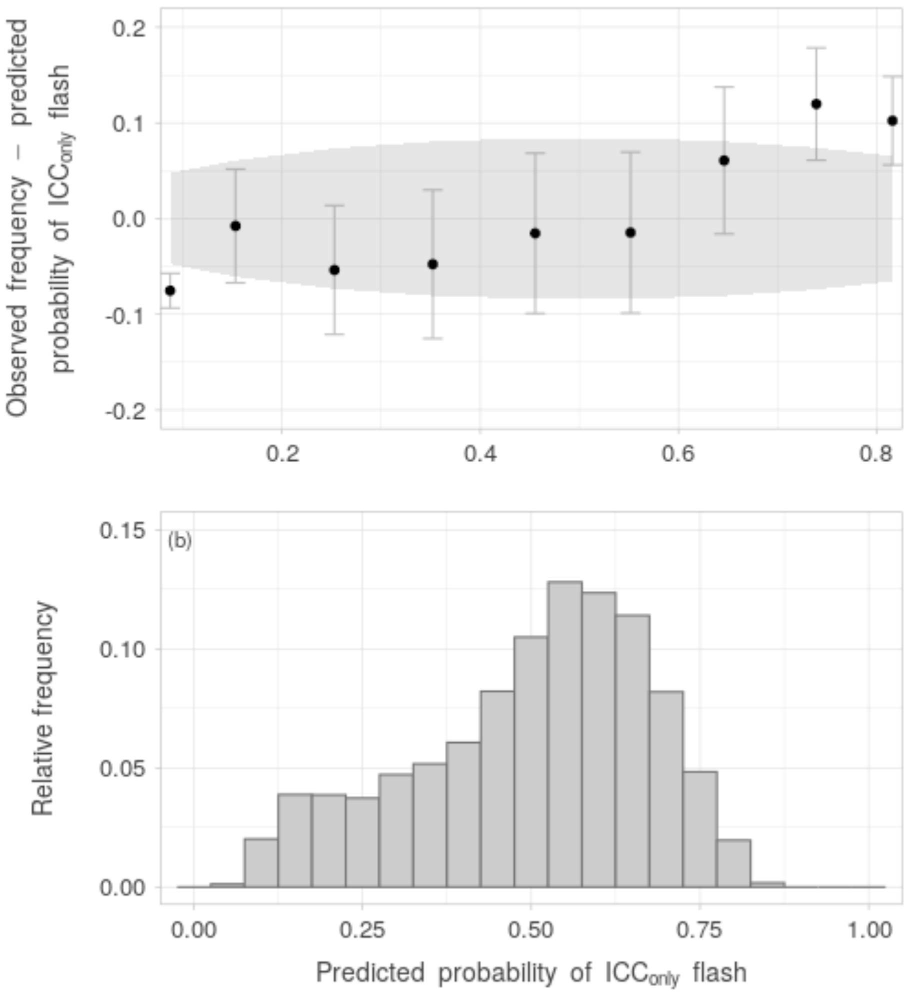

Figure S3. Panel (a): Difference of the average observed relative frequency and predicted probability and refinement distribution (b) for $\mathrm{ICC_{only}}$ versus $\mathrm{ICC_P} + \mathrm{ICC_{RS}}$ flash type models based on test data samples. Median of AUC is 0.75. Shaded area and error bars indicate the 95 % confidence interval around the average predicted probabilities and average observed frequencies, respectively.

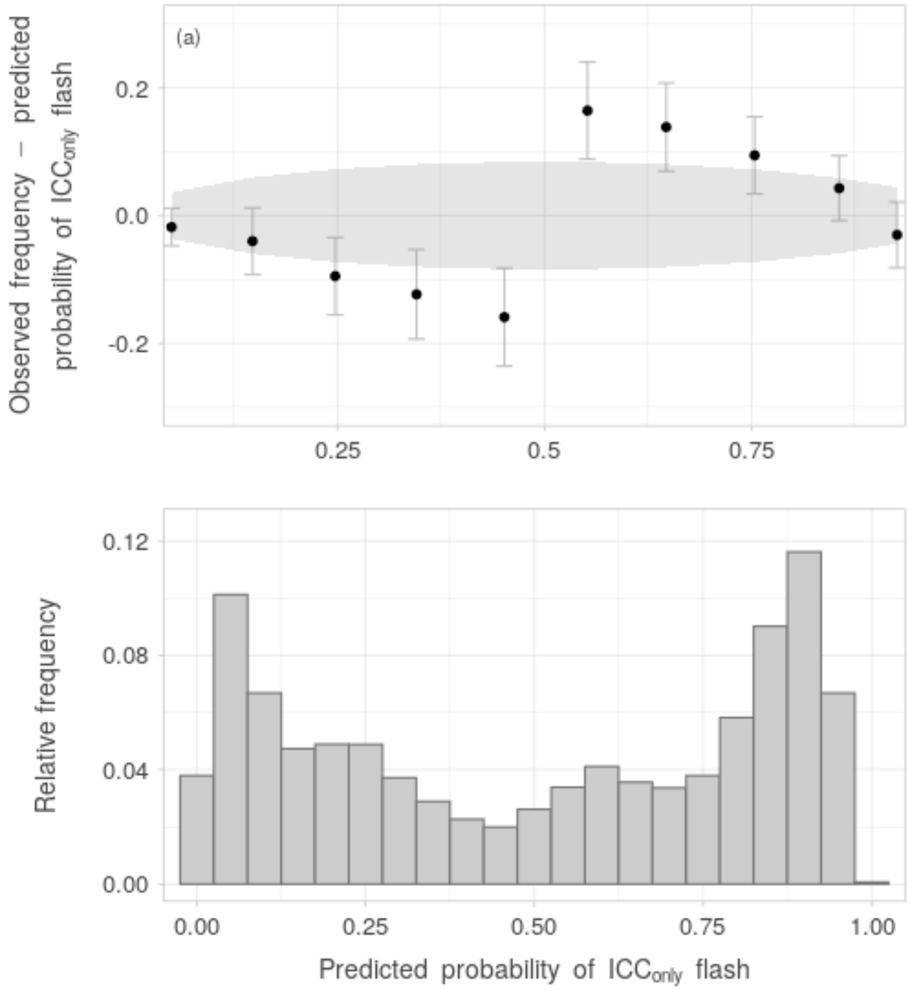

Figure S4. Panel (a): Difference of average observed relative frequency and predicted probability, (b): refinement distribution for $\mathrm{ICC_{only}}$ versus $\mathrm{ICC_P} + \mathrm{ICC_{RS}}$ flash type models when including information on narby lightning events (closest cloud-to-ground or intra-cloud lightning). The performance is based on test data samples. Median of AUC is 0.91. Shaded area and error bars indicate the 95 % confidence interval around the average predicted probabilities and average observed frequencies, respectively.

Table S1. Table of variables taken from ERA5 and variables derived from ERA5 assigned to different groups. The derived variables (indicated in italics) are subjectively chosen from meteorological knowledge to be potentially important in the charging process of a thundercloud or for the development of convection.

**Cloud physics**
cloud base height above ground
convective precipitation (rain + snow)
convective snowfall
high cloud cover
large scale precipitation
large scale snowfall
low cloud cover
maximum precipitation rate (rain + snow)
medium cloud cover
ice crystals (total column, tciw)
cloud droplets (total column, tclw)
raindrops (total column, tcrw)
Solid hydrometeors (total column, tcsw)
supercooled liquid water (total column, tcslw)
vertical integral of divergence of cloud frozen water flux
vertical integral of divergence of cloud liquid water flux
vertical transport of liquids around -10 C
ice crystals (-10 C - -20 C)
ice crystals (-10 C - -40 C)
ice crystals (-20 C - -40 C)

Cloud physics

cloud water droplets
(-10 C - -20 C)

cloud water droplets
(-10 C - -40 C)

cloud water droplets
(-20 C - -40 C)

raindrops
(-10 C - -20 C)

raindrops
(-10 C - -40 C)

raindrops
(-20 C - -40 C)

solid hydrometeors
(-10 C - -20 C)

solid hydrometeors
(-10 C - -40 C)

solid hydrometeors
(-20 C - -40 C)

solids (cswc + ciwc)
around -10 C

liquids (clwc + crwc)
around -10 C

cloud top height above ground

Moisture field

2 m dew point temperature

mean vertically integrated
moisture divergence

vertical integral of divergence
of moisture flux

vertically integrated moisture
divergence

water vapor
(-10 C - -20 C)

water vapor
(-10 C - -40 C)

water vapor
(-20 C - -40 C)

water vapor (total column)

Surface exchange

boundary layer height

evaporation

instantaneous surface
sensible heat flux

surface latent heat flux

net surface solar radiation

net surface thermal radiation

surface sensible heat flux

downward surface solar radiation

Temperature field

2 m temperature

skin temperature

convective available
potential energy

convective inhibition

mean sea level pressure

height of 0 C isotherm

height of -10 *C isotherm*

height of -20 *C isotherm*

mean sea level pressure difference (North to South)

mean sea level pressure difference (East to West)

mean sea level pressure difference (North-East to South-West)

mean sea level pressure difference (South-East to North-West)

Wind field

boundary layer dissipation

vertical integral of total
energy divergence

u component of wind at cloud base

v component of wind at cloud base

u component of wind at cloud top

v component of wind at cloud top

total cloud shear

total cloud shear direction

wind speed at cloud top

wind speed at cloud base

wind speed at 10 m

wind direction at cloud top

wind direction at cloud base

shear between 10 m and cloud base

shear between 10 m and cloud base (direction)

Climatological background

Day of year (starting from 01 July)

Hour of day (starting from 8 am)
